# Impact of Treatment Decentration on Higher-Order Aberrations after SMILE

**DOI:** 10.1155/2017/9575723

**Published:** 2017-03-15

**Authors:** Ying Yu, Wenwen Zhang, Xinliang Cheng, Jianru Cai, Hui Chen

**Affiliations:** ^1^Eye Institute, Affiliated Hospital of Nantong University, Nantong 226001, China; ^2^Nanjing Ning Yi Eye Center, Nanjing Drum Tower Hospital, Nanjing 210008, China

## Abstract

*Purpose*. To evaluate decentration following femtosecond laser small incision lenticule extraction (SMILE) and sub-Bowman keratomileusis (SBK) and its impact on higher-order aberrations (HOAs). *Methods*. Prospective, nonrandom, and comparison study. There were 96 eyes of 52 patients who received SMILE and 96 eyes of 49 patients who received SBK in this study. Decentration was calculated 6 months after surgery with Pentacam. HOAs and visual acuity after the surgery were examined for patients in both groups before and 6 months after surgery. *Results*. The mean decentration displacement in SMILE group was significantly less than SBK group (*P* = 0.020). 89 eyes were decentered within 0.50 mm after SMILE and SBK. The association between vertical decentration and the induced spherical aberration was insignificant in SMILE group (*P* = 0.035). There was an association between decentration and safety index, efficacy index, vertical coma, spherical aberration, and HOAs in root mean square (RMS, *μ*m) after SBK (all *P* < 0.05). No difference was found in uncorrected and corrected distance visual acuity, safety index, efficacy index, and wavefront aberrations between the two subgroups at any delimited value after SMILE (all *P* > 0.05). Decentration exceeding 0.37 mm affected vertical coma and RMSh of SBK eyes (*P* = 0.002, 0.005). *Conclusion*. SMILE surgery achieved more accurate centration than SBK surgery. Vertical decentration is associated with the induced spherical aberration in SMILE.

## 1. Introduction

Accurate centration plays an important role in corneal refractive surgeries. The ideal treatment center is the corneal intercept of the visual axis. Since it is difficult to center the crossing point, vertex normal-centered and pupil-centered ablations are commonly used in excimer laser surgeries. Although the active eye tracker and iris registration may decrease the incidence of decentration, decentered treatment still occurs clinically.

Decentered treatment is a common complication after corneal refractive surgery [1, 2] and plays a major role in the induction of higher-order aberrations (HOAs) [3]. In photorefractive keratectomy (PRK) surgery, decentration more than 0.30 mm was more likely to induce HOA, spherical aberration, and coma, as compared with the ablation decentration of less than 0.15 mm [4]. It was reported that eyes with well-centered ablations had a significantly lower magnitude of aberrations and better UCVA than eyes with decentered ablations after LASIK [5]. The induced HOAs may result in glare, halos, monocular diplopia, deterioration in visual performance, and subsequent patients' dissatisfaction.

Small incision lenticule extraction (SMILE) is a new surgery for the treatment of myopia and myopic astigmatism without corneal flap. Due to its excellent effect, safety, and predictability, SMILE has been widely accepted [6, 7]. However, there is no accurate centration and eye tracker during this treatment. The centration is operated by a surgeon during docking. When the surgical bed moves slowly toward the cornea, the vertex of the cornea may fit the center of the contact surface. Accurate centration is more dependent on a surgeon's experience. Also, there is no adjustment to maintain alignment with eye movement during the procedure. Therefore, it may increase decentration and induce more HOAs. The association between decentration and corneal surface wavefront aberrations (WFAs) during SMILE has not been well documented.

In this prospective study, we evaluated the distribution of optic zone decentration, uncorrected and corrected distance visual acuities (UDVA, CDVA), and HOAs following SMILE and sub-Bowman keratomileusis (SBK) for 6 months postoperatively. We also examined the relationship between HOA, visual quality, and decentration. Patients who received SMILE surgery during the learning curve were also included to further measure the impact on refractive outcomes from decentration.

## 2. Patients and Methods

### 2.1. Patients

This study was approved by the ethics committee of the Affiliated Hospital of Nantong University (Nantong, China). A total of 110 patients (214 eyes) undergoing surgeries for the correction of myopia and myopic astigmatism were recruited for the study at the Affiliated Hospital of Nantong University. Fifty-five patients (108 eyes) received SMILE and 55 patients (106 eyes) received SBK surgery, while there were 12 eyes in the SMILE group and 10 eyes in the SBK group who were lost to follow-up 6 months postoperatively. The clinical information is shown in Table 1. All recruited patients have read and signed an informed consent form in accordance with the Declaration of Helsinki.

The inclusion criteria were as follows: a minimum age of 18 years; a CDVA better than 20/25; a spherical equivalent between −1.5D and −10.0D within −3.0D of cylindrical diopter; stable refractive errors in the past 2 years; and without wearing soft contact lenses for 2 weeks and hard contact lenses for 4 weeks before the preoperative examinations. Exclusion criteria included keratoconus, corneal diseases, glaucoma active ocular or systemic diseases, and more than 0.4 mm of kappa angle. Before and after surgery, all patients received measurements of UDVA, CDVA, intraocular pressure (Topcon CT-80A, Tokyo, Japan), slit lamp microscope, fundus examination, manifest refraction, breakup time (BUT) of tear film, Schirmer test, corneal topography (Pentacam 70700: Oculus, Wetzlar, Germany), and HOAs.

### 2.2. Measurement of WFAs

Mesopic anterior corneal surface WFAs were measured before surgery and 6 months postoperatively with a Scheimpflug camera. Measurements were repeated three times and the best quality image (quality specification = OK) was used for analysis. Coefficients of the Optical Society of America standard for a 6.0 mm central diameter were analyzed. The root mean square (RMS, *μ*m) values were used to evaluate HOAs. The spherical aberration (Z_4_^0^), vertical coma (Z_3_^−1^), and horizontal coma (Z_3_^1^) were also analyzed.

### 2.3. Decentration Measurements

A front elevation map (in a one-map view) at 6 months after surgery was taken as confirmation of decentration from the corneal vertex. The (0, 0) point was shown as the corneal vertex and a black cross at the pupil center in the topographic graph. When the cursor moved, a coordinate (*x*, *y*) appeared for any point relative to (0, 0) point. The value of the thinnest point coordinate was determined as the vertical and horizontal displacement. The quadratic sum of the vertical and horizontal displacement was defined as *z*^2^ (the vector displacement). Hence, the vector displacement was the square root of the coordinate. The method was previously described [8].

### 2.4. Surgical Methods

All SMILE surgeries were performed with a femtosecond laser system (VisuMax; Carl Zeiss Meditec AG, Jena, Germany). According to a previous report [9], the pulse energy was 130 nJ with 500 kHz repetition rate. The thickness of the cap was set to 120 *μ*m. The diameter of the cap was 7.5 mm and the diameter of the refractive lenticule was 6.5 mm. A side cut made for access to the lenticule was set 120° apart in a circumferential width of 2.0 mm.

In the SBK group, Moria One-use plus (Moria, Antony, France) microkeratome was used to create a flap and flap hinge located in the nasal position. The central thickness of the flap was 110–120 *μ*m and the diameter of the flap was 8.5–9 mm. The following ablation was set as 6.5 mm of optic zone with 2.0 mm of a transition zone. After a flap was created, the flap was raised, the stromal bed was dried with a sponge, and then the ablation was performed with Allegretto Wave excimer laser (Lumenis, America) in a conventional treatment algorithm. When the ablation was completed, the flap was put back gently. A balanced salt solution was used to flush the cornea.

All surgeries were performed by a single surgeon (XLC). After the surgery, all patients were given 0.3% levofloxacin (Santen Inc., Japan), 0.1% fluorometholone (Santen Inc., Japan), and Tears Naturale Forte (Alcon Laboratories Inc.).

### 2.5. Data Analysis

Statistical analysis was performed using SPSS 19.0 statistical software (SPSS, IBM, USA). Considering the potential correlations between two eyes of a subject, a generalized estimating equation (GEE) model was used to the comparisons for the pre- and postoperative changes of WFAs. Assessing statistical differences between the subgroups, postoperative changes in two surgical groups were also was examined by GEE. Spearman's rank correlation test was used to assess the relationship between the decentration and the changes of all HOAs of the anterior corneal surface. A *P* value of less than 0.05 was considered statistically significant.

## 3. Results

### 3.1. Optical Zone Center Locations

The mean total decentration was 0.27 ± 0.09 mm (range, 0.01–0.76) in the SMILE group and 0.32 ± 0.14 mm (range, 0.02–0.89) in the SBK group at 6 months postoperatively. A significant difference was found between these two groups (*P* = 0.020). The horizontal, vertical, and vector displacements in decentration in SMILE and SBK surgeries are shown in Table 2. A significant difference was observed in the displacement of vertical decentration (*P* = 0.000) and in the horizontal decentration (*P* = 0.024) between two surgical groups.

**Figure 1:**
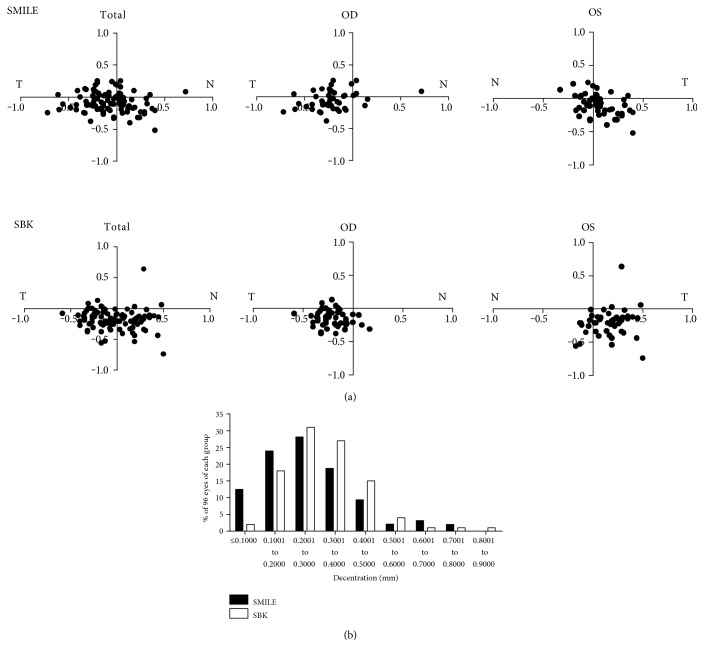
(a) Scatterplot showing the distribution of treatment centers (black dots) in relation to the corneal vertex in total eyes, right eyes, and left eyes of SMILE and SBK surgeries. Positive vertical coordinates stand for superior displacements and the negative for inferior ones. (b) Distribution of total decentered displacements (millimeters) in two surgeries.

Comparing the results between the right and left eyes, differences existed in the total decentered amount, horizontal decentration, and vertical decentration (*P* = 0.001, 0.000, and 0.000, resp.) after SMILE surgery. In SBK eyes, differences existed in both the vertical decentration and the total decentered amount (*P* = 0.000 and 0.000, resp.), but not in the horizontal decentration (*P* = 0.331) between the right and left eyes. Between the two groups, there were differences in the total decentered amount, the horizontal decentration, and the vertical decentration (*P* = 0.024, 0.000, and 0.000, resp.) (Table 2). Distributions of decentered displacements in two surgeries are shown in Figures 1(a) and 1(b).

All decentration displacements were within 0.9 mm. There were 89 SMILE-treated eyes (93%) and 89 SBK-treated eyes (93%) within 0.5 mm of decentration and 62 SMILE-treated eyes (65%) and 49 SBK-treated eyes (51%) within 0.3 mm.

### 3.2. Refractive Outcome

**Figure 2:**
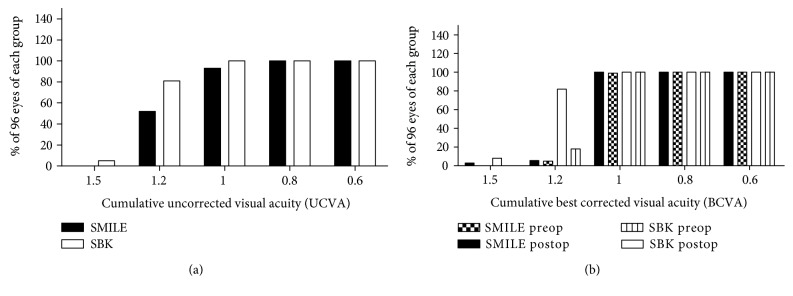
(a) Distribution of uncorrected distance visual acuity (UDVA) at cumulative 6 months postoperatively in the SMILE and SBK groups. (b) Distribution of corrected distance visual acuity (CDVA) at cumulative before and 6 months after surgeries in the SMILE and SBK groups.

**Figure 3:**
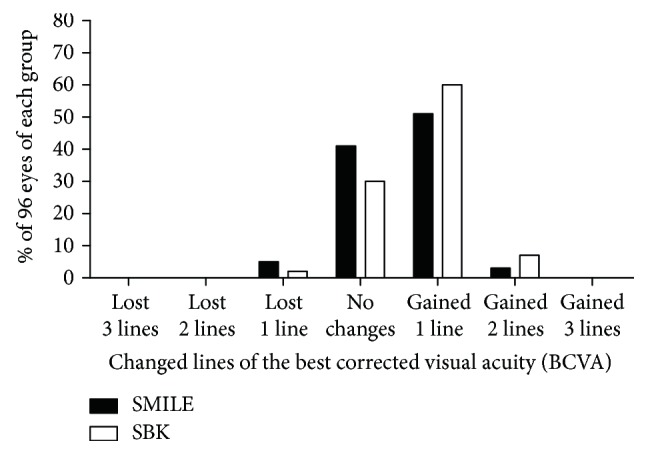
The percentage of the eyes with a gain/loss of Snellen CDVA lines compared with preoperative levels for different surgeries at 6 months postoperatively.

Six months after surgery, 93% of SMILE-treated eyes (89 eyes) and 100% of SBK-treated eyes (96 eyes) showed a UDVA better than 20/20. All treated eyes were better than 20/25. All eyes showed a CDVA better than 20/20 suggesting the safety of these two surgeries. Forty-eight SMILE- (50%) and 58 SBK- (60%) treated eyes gained one line; 3 SMILE- (3%) and 7 SBK- (7%) treated eyes gained two lines; and 5 SMILE- (5%) and 2 SBK- (2%) treated eyes lost one line (Figures 2(a), 2(b), and 3).

The safety index was 1.10 ± 0.14 in the SMILE group, less than that in the SBK group (1.15 ± 0.14, *P* = 0.006). The efficacy index was 1.07 ± 0.13 in the SMILE group, also less than that in the SBK group (1.14 ± 0.13, *P* < 0.001).

### 3.3. Wavefront Aberrations

**Figure 4:**
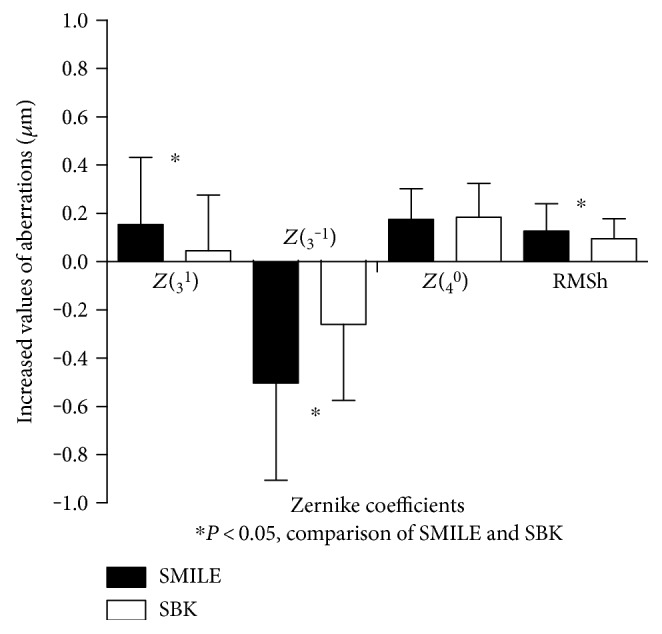
Changes in wavefront aberration before and 6 months after surgeries in the SMILE and SBK groups.

There were significant increases in horizontal coma in the SMILE group and vertical coma, spherical aberration, and RMS of HOAs in the two groups at 6 months after surgery (Wald χ^2^ = 24.504, 97.075, 140.708, 118.867, all *P* < 0.001). At 6 months postoperatively, the horizontal coma, vertical coma, and RMS of HOAs were higher in the SMILE group than in the SBK group (Table 3, Figure 4) (*P* = 0.002, *P* < 0.001, and *P* < 0.001).

### 3.4. Association Analysis

#### 3.4.1. Decentration and Wavefront Aberrations

By Spearman analysis, in the SMILE group, there was no correlation between horizontal displacement and WFAs. However, there was a positive correlation between vertical displacement and vertical coma and RMS of HOAs (*r* = 0.348, *P* = 0.242; *r* = 0.001, *P* = 0.017). A positive correlation was also detected between vector decentration and spherical aberration (*r* = 0.216, *P* = 0.035).

In the SBK group, there were positive correlations between the horizontal decentration and the horizontal and vertical coma (*r* = 0.386, *P* < 0.001; *r* = 0.238, *P* = 0.019). Vertical displacement positively correlated with vertical coma (*r* = 0.242, *P* = 0.017). There was also a positive correlation between vector decentration and vertical coma, spherical aberration, and RMS of HOAs (*r* = 0.384, *P* < 0.001; *r* = 0.229, *P* = 0.025; *r* = 0.366, *P* < 0.001).

#### 3.4.2. Vector Decentration Classification and Wavefront Aberrations

We have set up the median and quartile value to compare the effect on refractive outcomes by vector decentration between the two surgical groups (*P*_75_ = 0.37 mm, *P*_50_ = 0.27 mm, and *P*_25_ = 0.19 mm).

Taking 0.37 mm as the delimited value, there was no significant difference in WFAs after the SMILE surgery. The vertical coma and RMS of HOAs were higher in the subgroup with more than 0.37 mm of decentration than those within 0.37 mm in the SBK group (*P* = 0.002, 0.005).

Taking 0.27 mm as the delimited value, there was no significant difference in WFAs between the two subgroups in the SMILE surgery, while there was a significant difference in vertical coma, spherical aberration, RMS of HOAs, and safety and efficacy index (*Z* = −3.222, −2.218, −3.880, *P* = 0.001, 0.027, 0.000) between the two subgroups in the SBK group.

If 0.19 mm was taken as the delimited value, no difference in all refractive outcomes was detected between the two subgroups in the SMILE and SBK groups.

## 4. Discussion

Decentration is one of the serious postoperative complications after PRK and laser-assisted in situ keratomileusis (LASIK) [2, 10]. Decentration played a major role in increased coma and spherical aberrations after corneal refractive surgery [11]. Li et al. suggested that in the early learning curve of the SMILE surgery, although mild decentration occurred, good visual outcomes were achieved [8]. In this study, we compared the association between decentration and HOAs after SMILE and SBK.

In our study, the displacement of decentration in the SMILE group was less than that in the SBK group. Moreover, the percentage of decentration displacement within 0.3 mm was higher in the SMILE (65%) than in the SBK (51%) group. The horizontal displacement of decentration was higher after SMILE surgery than after SBK surgery. There was a significant difference between two surgeries in terms of vertical displacement of decentration. Therefore, we believe that SMILE surgery is able to achieve better centration especially in vertical direction as compared with SBK surgery. In addition, there was less vector displacement of decentration in the left eye than in the right eye after SMILE surgery. However, there was more vector displacement of decentration in the left eye than in the right eye after SBK surgery. It was speculated that patients felt more comfortable during the SMILE surgery procedure than the SBK surgery and therefore cooperated better.

The various displacements of decentration reported in previous studies were related to a different centration reference, surgical method, equipment, and analytical method. Lee et al. reported the displacement of decentration as 0.23 ± 0.10 mm after PRK [4]. Li et al. [8] reported that after SMILE surgery, the decentered displacements of all eyes were within 0.50 mm, with 70% eyes within 0.20 mm and 90% eyes within 0.30 mm. In the study of Lazaridis et al. [12], the decentration of lenticule from the pupil center was 0.326 ± 0.196 mm after SMILE surgery and 0.452 ± 0.224 mm after FS-LASIK surgery. In a recent article on SMILE surgery, the decentration to the pupil center was 0.07 mm (range, 0–0.17 mm) [13]. Cavanaugh et al. reported that 92.7% decentered level was within 1.0 mm and 52.7% within 0.5 mm in PRK [14]. Another study showed that 91.3% decentration was within 0.75 mm in excimer laser corneal refractive surgeries. The decentered amount within 0.75 mm was 93.9% in PRK and 88.7% in LASIK [15].

Our two surgeries all provided satisfactory results without serious complications. These results were similar to those of previous studies [16–19]. However, UCVA and CDVA were better in the SBK group than in the SMILE group. The reasons for the difference could be the different principles for surgeries, in which lenticule extraction in SMILE and stroma ablation with the wavefront aberration were optimized in SBK.

In our study, between horizontal displacement and HOAs, there was no correlation in the SMILE group but a positive correlation in the SBK group. However, the horizontal displacement in the SMILE group was similar to the value in the SBK group. Hence, we believe that SMILE is better tolerated for horizontal displacement than SBK. In the SMILE group, there was no correlation between horizontal displacement and HOAs but a positive correlation between vertical displacement and HOAs, and a positive correlation existed between vector decentration and HOAs. Therefore, the vertical displacement may have a greater impact than the horizontal displacement in HOAs for SMILE.

Some studies suggest that coma aberrations were affected by decentration after SMILE [8]. Moreno-Barriuso et al. held the opinion that decentration of the ablation pattern may generate 3rd-order aberrations after LASIK [20]. Vestergaard et al. [21] and Gertnere et al. [22] found that FLEx induced less spherical aberrations than FS-LASIK. One recent article assumed that the SMILE procedure may induce smaller changes in the ocular HOAs than the LASIK procedure [23]. However, some studies suggested that other reasons to explain the induced HOAs are because of a small decentration after SMILE [12, 24]. Wu and Wang found that coma aberrations of the anterior cornea significantly increased after the SMILE surgery and the change was correlated with the SE [24]. Kamiya et al. found that induced third-order aberrations were significantly correlated with SE in the eyes after LASIK but not after FLEx [3, 25].

We showed that decentration was associated with changed spherical aberration and RMSh in two groups (*P* < 0.05) and was also related to increased horizontal coma and increased value of vertical coma in SBK eyes (*P* < 0.05). Changed HOAs of two SMILE subgroups differed significantly at 0.37 mm and no statistical differences at 0.27 mm or 0.19 mm. Meanwhile, changed HOAs of two SBK subgroups differed significantly at all delimited values. Therefore, we can draw the conclusion that decentration had more influence on HOAs of SBK than of SMILE postoperatively. In the procedure of SBK, the amount should be controlled within 0.27 or smaller. It is possible that the SMILE procedure may be better tolerated for treatment decentration; videlicet, a certain degree of decentered zone might have only a slight effect on patients' refractive outcomes.

A limitation in our study is that the 6-month follow-up period may not be sufficient to fully assess the impact of decentration on HOAs. Further studies are needed to better understand the impact of decentration on visual outcomes.

In conclusion, the results of this study suggest that the SMILE surgery can achieve more accurate centration than the SBK surgery. Although mild decentration occurred in SMILE and SBK, good visual outcomes were achieved after these two surgeries. It is necessary to minimize the decentration to decrease the induced spherical aberration.

## Figures and Tables

**Table 1:** The represented demographic data for the patients in our study (mean ± SD).

Preoperation	SMILE (96 eyes)	SBK (96 eyes)
Age	23.38 ± 4.99	23.32 ± 4.61
Patients (male/female)	24/28	24/25
BCVA	1.01 ± 0.05	1.03 ± 0.08
Spherical diopter	−5.16 ± 1.66	−4.85 ± 1.64
Cylindrical diopter	−0.70 ± 0.58	−0.72 ± 0.54
Spherical equivalent (SE)	−5.51 ± 1.70	−5.31 ± 1.67
Thinnest corneal thickness (*μ*m)	551.99 ± 33.33	542.98 ± 60.40

**Table 2:** The represented horizontal, vertical, and vector displacements in decentration in SMILE and SBK surgeries.

		Horizontal (mm)	Vertical (mm)	Vector (mm)
Total eyes	SMILE (*n* = 96)	−0.08 ± 0.25^∗^	−0.08 ± 0.16^∗^	0.27 ± 0.15^∗^
	SBK (*n* = 96)	−0.02 ± 0.24^∗^	−0.18 ± 0.17^∗^	0.32 ± 0.14^∗^

Right eyes	SMILE (*n* = 47)	−0.22 ± 0.23^†^	−0.05 ± 0.14^†^	0.31 ± 0.16^†^
	SBK (*n* = 48)	−0.20 ± 0.15	−0.15 ± 0.12^#^	0.30 ± 0.11^#^

Left eyes	SMILE (*n* = 49)	0.06 ± 0.17^†^	−0.11 ± 0.16^†^	0.23 ± 0.14^†^
	SBK (*n* = 48)	0.16 ± 0.17	−0.21 ± 0.20^#^	0.34 ± 0.16^#^

^∗^Generalized estimating equation (GEE) model comparison between SMILE and SBK, *P* < 0.05.

^†^GEE model comparison between the right and left eyes in the SMILE group, *P* < 0.05.

^#^GEE model comparison between the right and left eyes in the SBK group, *P* < 0.05.

The positive value stands for nasal and superior direction relative to pupil center.

**Table 3:** Comparison of the wavefront aberration between two surgeries 6 months postoperatively.

Wavefront aberrations	Surgeries	Results (*μ*m)	Wald *χ*^2^	*P*
Horizontal coma	SMILE	0.163 ± 0.304	9.630	0.002
	SBK	0.058 ± 0.280		

Vertical coma	SMILE	−0.542 ± 0.434	18.109	0.000
	SBK	−0.313 ± 0.323		

Spherical aberration	SMILE	0.420 ± 0.119	0.035	0.851
	SBK	0.423 ± 0.137		

RMS of HOAs	SMILE	0.278 ± 0.086	16.089	0.000
	SBK	0.233 ± 0.069		
